# Simultaneous transcriptome analysis of oil palm clones and *Phytophthora palmivora* reveals oil palm defense strategies

**DOI:** 10.1371/journal.pone.0222774

**Published:** 2019-09-25

**Authors:** Kelly Avila-Mendez, Ávila Rodrigo, Leonardo Araque, Hernán Mauricio Romero

**Affiliations:** 1 Biology and Breeding Program, OiI Palm Research Center, Cenipalma, Bogotá, Colombia; 2 Department of Biology, Universidad Nacional de Colombia, Bogotá, Colombia; Franklin & Marshall College, UNITED STATES

## Abstract

*Phytophthora palmivora* is an oomycete that causes oil palm bud rot disease. To understand the molecular mechanisms of this disease, palm clones with contrasting responses (Ortet 34, resistant and Ortet 57, susceptible) were inoculated with *P*. *palmivora*, and RNAseq gene expression analysis was performed. The transcriptome was obtained by sequencing using Illumina HiSeq2500 technology during the asymptomatic phase (24, 72 and 120 hours postinfection, hpi). A simultaneous analysis of differentially expressed gene (DEG) profiles in palm and *P*. *palmivora* was carried out. Additionally, Gene Ontology (GO) and gene network analysis revealed differences in the transcriptional profile of the two ortets, where a high specificity of the pathogen to colonize the susceptible ortet was found. The transcriptional analysis provided an overview of the genes involved in the recognition and signaling of this pathosystem, where different transcription factors, phytohormones, proteins associated with cell wall hardening and nitrogen metabolism contribute to the resistance of oil palm to *P*. *palmivora*. This research provides a description of the molecular response of oil palm to *P*. *palmivora*, thus becoming an important source of molecular markers for the study of genotypes resistant to bud rot disease.

## Introduction

The vegetable oil obtained from the cultivation of oil palm (*Elaeis guineensis* Jacq.) is produced at the lowest cost due to the high yield of the palms. Oil palm can produce 5 to 10 times more oil than other oil crops [1]. The production of palm oil originates mainly from 5 countries: Malaysia, Indonesia, Thailand, Nigeria and Colombia; in 2016, Malaysia and Indonesia accounted for 77% of the world's share of palm oil production [2].

Oil palm crops are affected by various diseases. In Latin America, the main cause of crop losses is the oomycete *Phytophthora palmivora* [3, 4]. According to Cenipalma´s (Colombian Oil Palm Research Center) Plant Health Management Program, in 2017, 75,000 hectares were affected by the disease, which caused losses of more than USD 1,800 million in the four oil palm-growing areas of the country.

Fungi and oomycetes can cause the most devastating diseases in agriculture [5]. Among the oomycetes, the genus *Phytophthora* is very important because some of the worst crop diseases are caused by members of this genus [6, 7]. *Phytophthora* species have a hemibiotrophic life cycle, in which the first part of their development is biotrophic (the cells stay alive and the plant does not show symptoms) followed by a necrotrophic phase in which the pathogen has internally colonized host cells and the plant shows symptoms due to the death of the cells [8].

*P*. *palmivora* is a hemibiotrophic organism that causes diseases in various tropical crops, such as papaya, cocoa, coconut and durian, among others [9]. Since its identification in 2008 as the causal agent of oil palm bud rot, studies have focused on the development of inoculation methods in immature leaflets where the initial symptoms of the disease have been replicated [10, 11]. However, the underlying basis of the molecular response of oil palm to bud rot disease has not been studied. Understanding the molecular bases of the oil palm-*P*. *palmivora* interaction will not only allow a description of the mechanisms associated with the interaction but will also provide the possibility of developing mechanisms for the early detection of resistant materials in oil palm breeding programs.

The main objective of this research was to obtain information on the molecular mechanisms associated with oil palm resistance to *P*. *palmivora*. A transcriptomic strategy was developed to elucidate the mechanisms of palm resistance and pathogen infection. This information will refine the search for genotypes resistant to the disease and understanding of how the pathogen attacks the palm. This work provides the first description of the molecular mechanisms by which oil palm responds to infection by *P*. *palmivora*.

## Materials and methods

### Plant material

Oil palm clones were produced by a somatic embryogenesis technique in the Tissue Culture Laboratory of the Colombian Oil Palm Research Center (Cenipalma); the process to obtain clones takes three years. The produced shoots were then placed on rooting media (MS media) for four months. At this point, the clones were inoculated with *P*. *palmivora*. At the time of inoculation, the clones were 10 cm tall and had three to five leaves.

The original explants were obtained from resistant (Ortet 34) and susceptible (Ortet 57) *Elaeis guineensis* donor palms. Ortet 34 was a Deli x Cameroon palm selected from the oil palm-growing area of Puerto Wilches (Santander, Colombia), an area that had suffered an outbreak of bud rot disease. Ortet 57 was an Angola *pisifera* palm selected from the Palmar de la Vizcaína Experimental Field.

The contrasting responses of the oil palm clones were determined using previously performed screening tests [12]. These tests showed statistically significant differences between the two ortets. Additionally, the behavior of the clonal materials was consistent with the behavior of the donor palms in the field.

### Pathogen

Cultures of an isolate of *P*. *palmivora* from a collection of the Plant Pathology Laboratory of the Colombian Oil Palm Research Center–Cenipalma- (CPPhZC-05) were used. The isolate was inoculated on Petri dishes in clarified V8 juice medium (20% V8 juice, 5 g/l CaCO3, 50 mg/l B-sitosterol, 1.5% agar, rifampicin 1 μg/mL). The pathogen was kept under dark conditions for the first 3 days at 25ºC. After three days, the Petri dishes were placed under a 12-hour photoperiod until the cultures completed 10 days of growth.

### Inoculation

The inoculations were made with 10-day-old cultures of *P*. *palmivora*. The *in vitro* clones were inoculated at the petiole base with 20 μL of a zoospore suspension at a concentration of 3x10^6^ zoospores/mL. The release of zoospores was performed by thermal shock with sterile distilled water at 4ºC, and the quantification of the pathogen was carried out using a Neubauer Chamber.

The plants were kept in a Weiss® growth chamber at a temperature of 28ºC with a photoperiod of 12 hours of light (72% relative humidity) and 12 hours of dark (75% relative humidity). Eight individuals of the susceptible ortet (Ortet 57) and eight individuals of the resistant ortet (Ortet 34) were inoculated per replication (two biological replicates). Three individuals per ortet were used for the negative control (mock inoculation), which were inoculated with 20 μL of sterile distilled water.

### Extraction of total RNA

Tissue from the petiole base of the palm clones was collected from 8 individuals per ortet per biological replicate for each time of infection (24, 72 and 120 hours post infection). RNA was extracted using the Ambion® (AM1912) kit (Ambion Inc., Foster City, Ca), following the manufacturer’s instructions.

For each ortet and time of infection, RNA pools were made for the construction of libraries. The libraries were prepared using the Truseq Stranded mRNA LT Sample Kit, and the samples were sequenced using the HiSeq2500 sequencing platform (126 bp paired-end sequences).

### Bioinformatics analysis

The cleaning of the reads obtained from raw data was performed by removing i) low quality reads (Q≥30) and ii) adapter sequences using Trimmomatic software (Version 0.36).

After the quality parameters of the reads of each sample were confirmed, the reads were mapped against the reference genome of *Elaeis guineensis* using STAR software (Version 5.3a). The bam files for subsequent analyses were obtained using Samtools (Version 1.6–4).

The reads of inoculated samples not aligned to the palm genome were mapped against a *P*. *palmivora* reference genome (http://www.ncbi.nlm.nih.gov/bioproject/558734, obtained from a high quality genome assembly based on long and short read sequencing [13]. The platforms Illumina (Illumina, Inc., San Diego, CA) and PacBio (Pacific Biosciences of California, Inc.) were used for sequencing. 2x150 bp paired-end libraries were obtained for Illumina; and for PacBio, libraries were designed to obtain 50X sequencing depth. The PacBio long reads were assembled de novo using the software Canu v1.7. The alignment of the Illumina reads was carried out with bowtie2 v2.3.4.1. Following the assembly of the reference genome, its structural annotation was performed using the tool MAKER.

Differential expression analysis was performed using the statistical package DeSeq 1.18.0–1 (Debian Package). The statistical parameters were an FDR (false discovery rate) cutoff of 0.05 and a fold change (FC) Log2> | 2 |. The identification of unique and overlapping genes of the differentially expressed gene dataset was performed using R-Venn 3.4.3–1 and R-Bioconductor 2.38.0–1.

For the functional analysis, the lists of differentially expressed genes (DEGs) were analyzed with Blast2GO, which generated a GO list for later analysis of networks.

For the network analyses we used the GO list with its p-value for each ortert and infection time and these data were exported to Revigo (REVIGO is freely available at http://revigo.irb.hr/). The information was loaded to the platform and using the interactive graph interphase the .xgmml files were obtained (these files can be used offline). The subsequent visualization of each network was performed using Cytoscape software Version 3.6.0 (https://cytoscape.org/). Networks were based on a color scale (preloaded by default in Cytoscape), the most significant nodes were highlighted, where the red nodes had greater control and/or contribution to the network formed.

### Gene validation by qRT-PCR

Twenty genes for the oil palm and four genes for *P*. *palmivora* were selected from the dataset to be validated by qRT-PCR. RNA was obtained from the inoculated and negative control clones of the contrasting ortets. First, the DNA was removed using the Ambion® DNAase-free Kit (Cat No AM1906), and the synthesis of the cDNA was carried out using the SuperScript IV First-Strand Synthesis System (Cat No 18901050), following the manufacturer’s instructions. The concentration of total RNA required for cDNA synthesis was 1 μg.

The qRT-PCR reactions were normalized with the GADPH (glyceraldehyde 3- phosphate dehydrogenase) gene for the oil palm qRT-PCR reactions, and a ribosomal protein gene was used for *P*. *palmivora*. For both genes, the expression value was verified against the obtained dataset. The primers used for the GADPH gene were those previously reported [14], and for the pathogen, the primer is listed in S1 Table. The qRT-PCR reactions were performed using a LightCycler 480 from Roche ®.

Fold change (FC) values of the qRT-PCR reactions were calculated using the Livak method [15]. RNA-seq FC, and qRT-PCR values were compared by linear regression analysis. The significance of the data was confirmed by Pearson`s rank correlation coefficient (after verifying the normality of the data) using Stata 14 software.

## Results

### Bioinformatics analysis—Mapping

After the cleaning of raw data, all the samples for each replicate showed a sufficient quantity of reads for the analysis (Table 1); an average of 120 million reads (clean data) was obtained for all samples, and more than 85% of the reads had a Q> 30, ensuring the quality of the sequencing process and the quality of the final reads obtained for the subsequent differential expression analysis.

**Table 1:** Reads, quality and mapping percentage of transcriptomic data.

Sample	Total Reads	Q30 (%)	Total Reads	Q30 (%)	Clean Data		Mapping (%) to *E*. *guineensis*	
	R1	R2	R1	R2	R1	R2	R1	R2
PP**	139.985.302	89,18	156.878.582	91,08	138.912.354	140.718.452	NA	NA
57-M*	125.166.670	91,42	140.518.938	90,51	124.557.916	126.728.540	88,36	88,91
34-M*	110.469.322	91,13	137.478.762	90,68	103.121.416	123.995.868	86,55	89,01
34–24	140.241.014	91,33	164.413.298	90,59	131.246.146	142.624.000	88,44	86,82
34–72	119.777.592	91,43	161.604.790	90,52	114.508.992	132.700.618	88,87	89,08
34–120	122.624.216	91,98	148.150.018	90,61	113.685.612	111.282.680	88,07	86,58
57–24	130.914.960	91,66	134.203.422	90,73	112.635.558	134.919.498	88,37	84,97
57–72	122.317.422	90,78	127.907.572	89,06	121.831.744	121.055.176	87,71	88,48
57–120	126.242.636	91,35	126.459.406	90,18	118.566.968	114.939.380	88,44	89,63

**PP: *P*. *palmivora*

*Mock Inoculation N/A not mapping realized, the sample correspond to a P. palmivora under growth in vitro conditions

The percentage of mapping performed with the *E*. *guineensis* genome and mapping values over 85% were obtained for all samples (Table 1), where 27000 of the 37000 genes of the genome were mapped and used in the subsequent differential expression analyses.

### Differential expression analysis

After the read quality verification process and the mapping of each sample were completed, the reads were used for differential expression analysis, which was performed independently for the plant reads and the pathogen reads.

**Fig 1:**
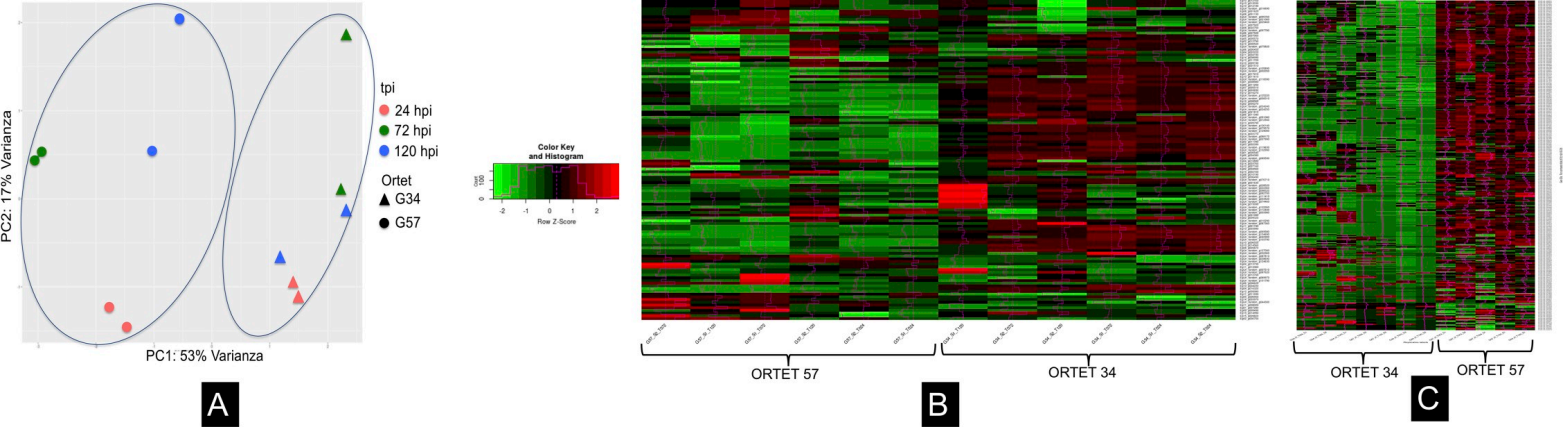
Differential expression profile for pathosystem oil palm clon- *P*. *palmivora*. A: Component Principal Analysis (PCA). B: Heatmap for oil palm clones C: Heatmap for *P*. *palmivora* expressing during infection process. Ortet 57: susceptible. Ortet 34: resistant.

The differential expression analysis (Fig 1) summarizes the expression profile found in the pathosystem evaluated. Principal component analysis (PCA) showed a differential distribution of the genes found per ortet and time of infection (Fig 1A), indicating that for each ortet and time of infection, the plant responded differently to the interaction with the pathogen.

Heatmaps showed different sets of gene clusters for the plant (Fig 1B), with differential expression patterns for each ortet, indicating that once the interaction with the pathogen started, the signaling cascades between the two ortets were different for the same gene.

Regarding the pathogen gene expression profile (Fig 1C), a large cluster was detected, indicating that the genes used by the pathogen to infect a susceptible ortet and a resistant ortet exhibited a completely different expression pattern.

**Fig 2:**
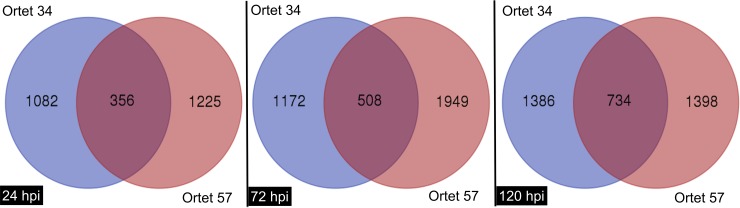
Venn diagram for unigenes expressing differentially in contrasting ortets. A. 24 hpi; B. 72 hpi; C. 120 hpi. Ortet 57: susceptible. Ortet 34: Resistant.

The Venn diagram analysis showed that out of the 37,000 genes in the palm genome, approximately 8,000 genes were expressed as unique genes for each ortet and time of infection for the conditions of this biological model (Fig 2).

The unique genes were used to perform the functional and network analyses to identify the different molecular strategies used by resistant or susceptible ortets to respond to infection by the pathogen.

#### Functional annotation

The genes obtained from the differential expression analysis dataset were used to carry out functional annotation for each main GO group: Molecular Function (GO: 0003674); Biological Process (GO: 0008150) and Cell Component (GO: 0005575).

**Fig 3:**
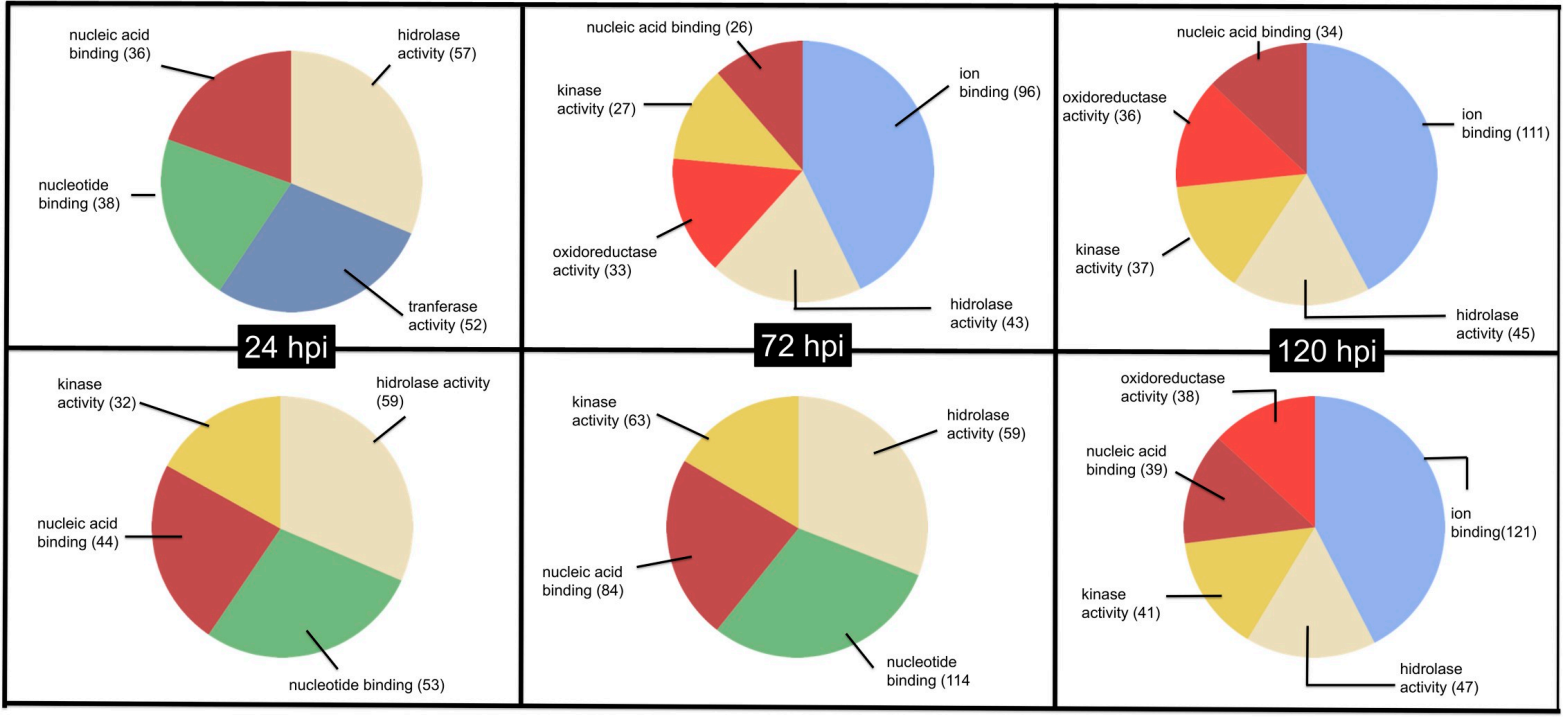
GO distribution for molecular function of DEG in oil palm clones during interaction process with *P*. *palmivora*. Top panel Ortet 34 (Resistant), Bottom panel Ortet 57 susceptible.

Regarding molecular function, in the three infection periods, a different GO distribution was found in the evaluated ortets; some of these were found as unique GOs for each material; for example, at 24 hours of infection, genes in the transferase category were found in the resistant ortet, while genes in the kinase category were found in the susceptible ortet (Fig 3).

At 72 hours post infection, the susceptible ortet maintained an equal distribution of its genes for this GO category, while the resistant ortet showed new categories of genes such as genes associated with the expression of kinase-like proteins, production of enzymes from oxidoreductase activity, and ion-binding proteins. Finally, at 120 hpi, a similar distribution in the GO of the molecular function was found in the two ortets.

**Fig 4:**
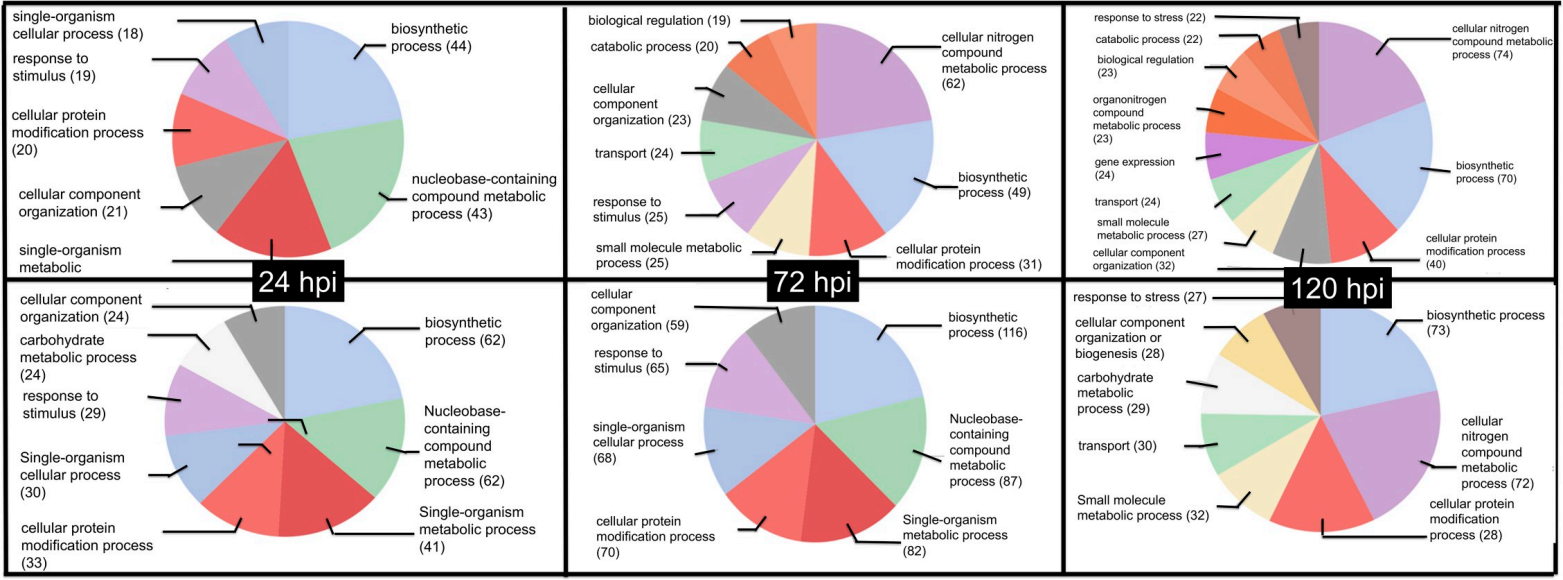
GO distribution for biological process of DEG in oil palm clones during interaction process with *P*. *palmivora*. Top panel Ortet 34 (Resistant), Bottom panel Ortet 57 susceptible.

The GO associated with biological processes included the stress response category, which was found in both ortets. The main differences for this GO were related to other processes. Thus, at 24 hours post infection (hpi), the susceptible ortet showed a category associated with the metabolism of carbohydrates, while in the resistant material, the genes associated with the expression of nitrogen proteins were active at 72 hpi, a pathway that remained active until 120 hpi (Fig 4).

**Fig 5:**
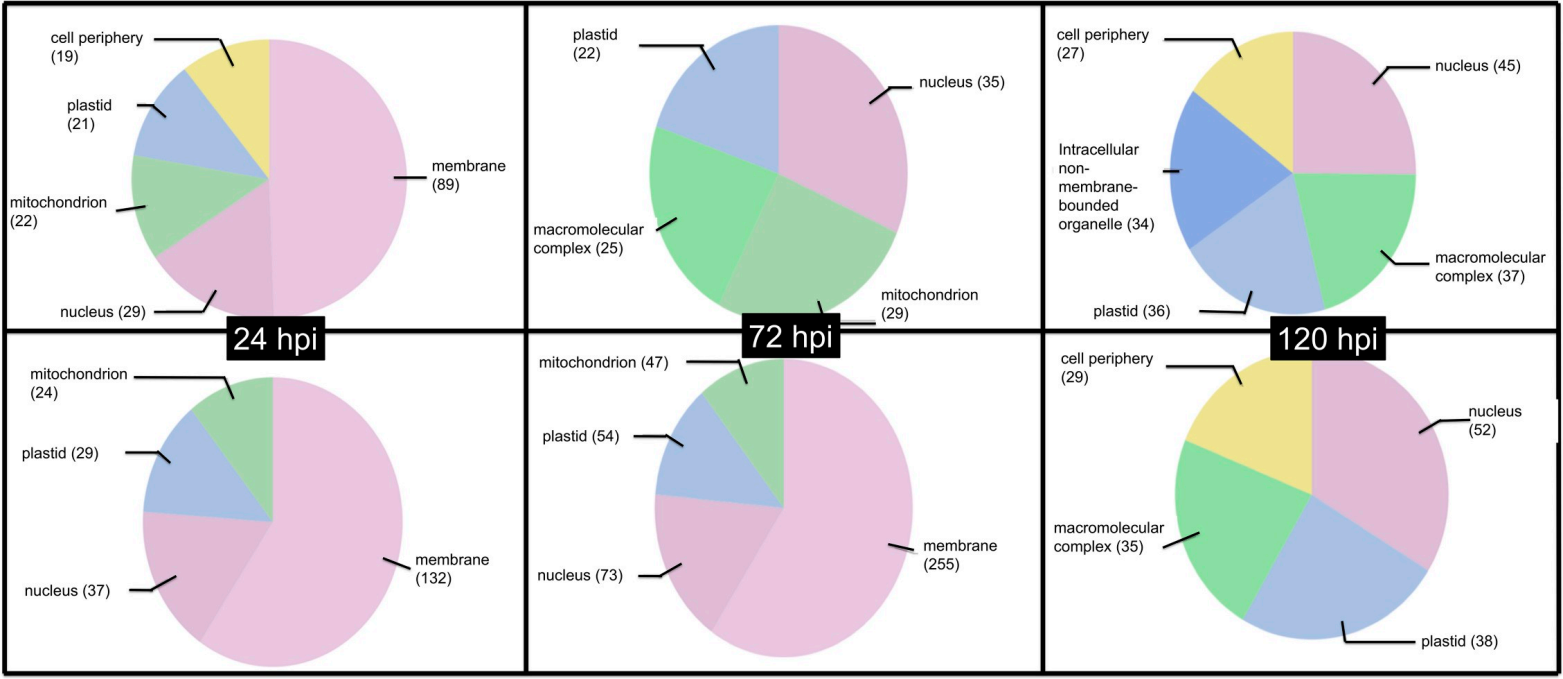
GO distribution for cellular component of DEG in oil palm clones during interaction process with *P*. *palmivora*. Top panel Ortet 34 (Resistant), Bottom panel Ortet 57 susceptible.

Finally, regarding the GO of the cellular component, a higher proportion of genes in the cell membrane was found for the susceptible ortet, which was maintained at 24 and 72 hpi, while for the resistant ortet, there was a greater distribution of the location of the genes in the different organelles in the 3 infection periods (Fig 5). These results suggest that from the first hours of infection, the two ortets perceived the pathogen, but molecular communication channels were established only with the susceptible ortet, which explains the differences observed in each category analyzed.

#### Network analysis

The distribution of the genes in the different GO categories made it possible to visualize the main differences between the susceptible and resistant ortets. To describe the palm-*P*. *palmivora* interaction model, we decided to generate networks associated with the GOs of the biological process (this category included the response to stress). In this way, it was possible to identify differences in the defense responses of the evaluated ortets.

**Fig 6:**
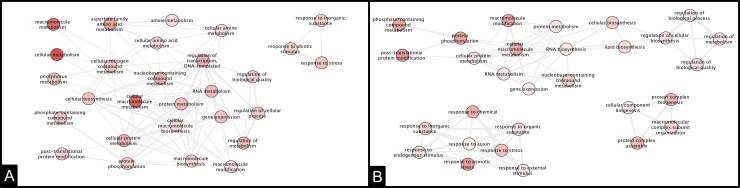
Interaction network of DEG in oil palm clones at 24 hpi of infection. A: Ortet 34 resistant B: Ortet 57 susceptible.

At 24 hpi, both ortets showed differences in the networks. For the resistant ortet, two main networks were identified with three principal nodes (Fig 6A) associated with the macromolecule and cellular metabolism, while in the susceptible ortet, the networks did not have principal nodes established during this infection period (Fig 6B).

At 72 hpi, the network configuration changed significantly for both ortets, where principal nodes were detected. However, each ortet formed different molecular networks, which demonstrates the contrasting response of the ortets to *P*. *palmivora*.

**Fig 7:**
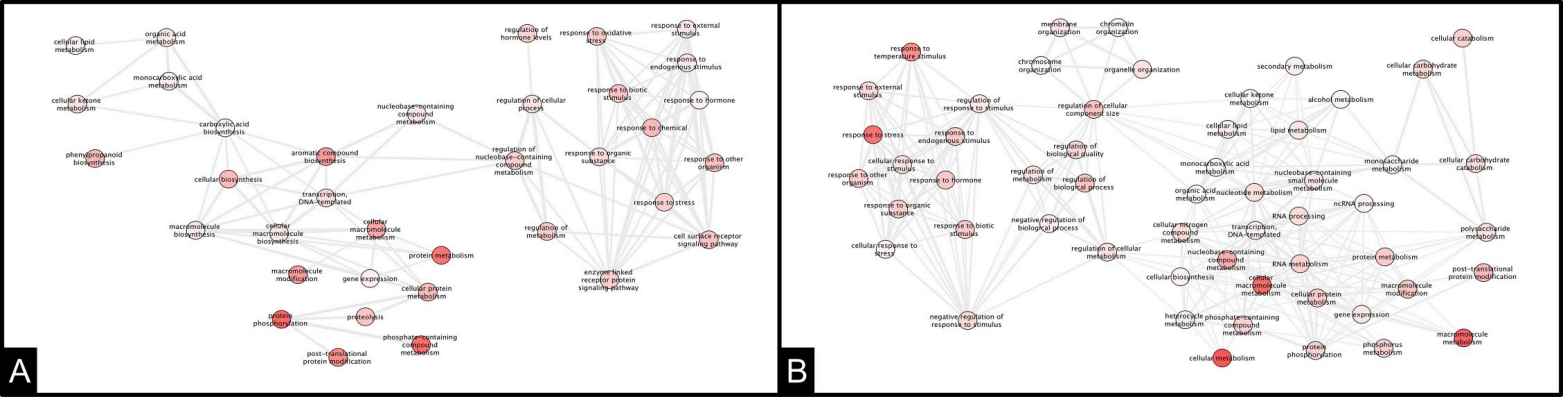
Interaction network of DEG in oil palm clones at 72 hpi of infection. A: Ortet 34 resistant B: Ortet 57 susceptible.

The resistant ortet (Fig 7A) developed networks associated with the metabolism of the phenylpropanoid pathway, protein metabolism, protein phosphorylation and phosphate-containing compound metabolism. In contrast, the susceptible ortet (Fig 7B) showed a more complex network, with a principal node related to the response to stress, which was connected with a large number of nodes, one of them related to the negative regulation of the response to stimulus.

**Fig 8:**
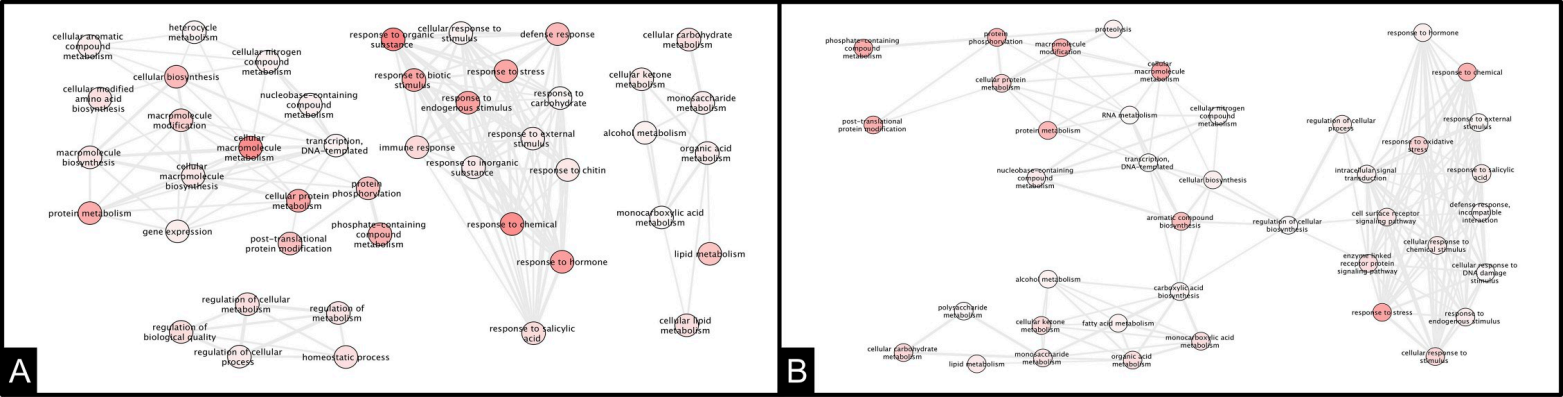
**Interaction network of DEG in oil palm clones at 120 hpi of infection** A: Ortet 34 resistant B: Ortet 57 susceptible.

At the end of the biotrophic phase, each ortet continued to show large differences in the networks activated. The resistant ortet (Fig 8A) finished this phase with four networks, and a new one was formed related to homeostatic regulation. For the susceptible ortet (Fig 8B), only one network was present: the process related to the response to stress. However, principal nodes were not found at this time of infection.

### Gene validation

The selected genes for oil palm are reported in Table 2. These genes had a relationship with some of the different defense pathways in plants. For example, genes related to the reinforcement of the cell wall, such as chorismate synthase (CHO), wall-associated receptor kinase (WAK),

**Table 2:** List of validated genes for q-RTPCR.

No	GenID	Candidate Gene Name
1	Eg14_g010350	Chorismate synthase
2	Eg11_g000710	Wall-associated receptor kinase
3	Eg12_g002670	Caffeoylshikimate esterase
4	Eg04_g024000	WRKY transcription factor 40
5	Eg08_g012810	1-aminocyclopropane-1-carboxylate oxidase
6	Eg11_g007120	MYB transcription factor MYB92
7	Eg04_g002030	GATA transcription factor
8	EgUn_random_g095760	Acidic endochitinase-like
9 y 10	Eg06_g004420—Eg08_g013050	Disease resistance protein
11	Eg04_g002650	Transport inhibitor response 1 like
12	Eg08_g009900	Callose synthase
13	EgUn_random_g124950	Pathogenesis-related protein
14	Eg15_g015620	Immediate early response 3-interacting protein 1
15	Eg08_g003440	CTP synthase
16	Eg04_g024650	Ethylene insensitive 3
17	Eg09_g008080	1-phosphatidylinositol-3-phosphate 5-kinase
18	Eg02_g011620	Transcription factor bHLH
19	Eg13_g003470	MLO
20	EgUn_random_g049390	Glucan endo-1,3-beta-glucosidase-like
21	PpalZC1_00000025-RA	Ribosomal protein
22	PpalZC1_00032933-RA	Elicitin
23	PpalZC1_00000691-RA	Protease
24	PpalZC1_00005681-RA	Sporulation
25	PpalZC1_00006753-RA	Thioredoxin

callose synthase (CALLO), and caffeoyl shikimate esterase (CAF) (Fig 9), were overexpressed in the resistant ortet.

The susceptible ortet showed overexpression of genes such as acidic endochitinase (AEL), pathogenesis-related protein (PR-1) and glucan endo-1,3-beta-glucosidase (GEB), genes that although involved in defense responses, failed to trigger an effective response against the pathogen advance. Additionally, at 24 hpi, this ortet showed overexpression of Mildew Resistance Locus O (MLO), a gene associated with susceptibility.

The genes selected for *P*. *palmivora* were: a gene related to sporulation process and, three genes related to pathogenesis, elicitin, protease and thioredoxin. In the four selected genes, the FC was over 2 when P. palmivora was inoculated on the susceptible clones. However, the FC of the same genes did not reach the FC threshold of 2 when the pathogen interacted with resistant clones (S1 Fig)

A Pearson correlation was conducted for the genes validated for oil palm and the pathogen. The obtained qRT-PCR values were correlated with the FC values obtained from the DEG data set. The Pearson correlation for the oil palm genes was 0.83, and for the pathogen genes, it was 0.87

## Discussion

In this study, Illumina (HiSeq2500) technology was used to explore and describe the contrasting responses of oil palm clones to inoculation with *P*. *palmivora*. Currently, the molecular mechanisms of the defense response in the *oil palm-P*. *palmivora* interaction process are not known. The next two sections will include the analysis related to oil palm defense and *P*. *palmivora* pathogenicity strategies.

### Oil palm molecular response

Previous studies conducted by our research team found that the biotrophic (asymptomatic) phase of the disease in oil palm clones lasts approximately 120 hours [12]. The transcriptomic analysis showed differences between the two evaluated ortets, where a coordinated series of molecular responses between the palm and *P*. *palmivora* were observed.

With the GO enrichment analyses, we found differences in the response to the pathogen between the evaluated ortets. Thus, the differential expression of genes encoding kinase-type proteins found in the susceptible ortet and in the pathogen for the same infection period (24 hpi) suggests the establishment of signaling cascades necessary for successful colonization, as in other pathosystems in which effectors that interact with kinase-type proteins have been reported to block the defense response of plants [16].

An interesting result was TIR1 (TRANSPORT INHIBITOR RESPONSE 1) overexpression in the susceptible ortet (Fig 9), a response that has been observed in other pathosystems in which TIR overexpression has been related to pathogen susceptibility [17, 18]. In fact, auxins have been related to susceptibility to biotrophic pathogens [19], with TIR1 playing a major role. Thus, the reduction of TIR1 transcript levels through posttranscriptional gene silencing or downregulation via the salicylic acid pathway [20], increases pathogen resistance [21, 22].

**Fig 9:**
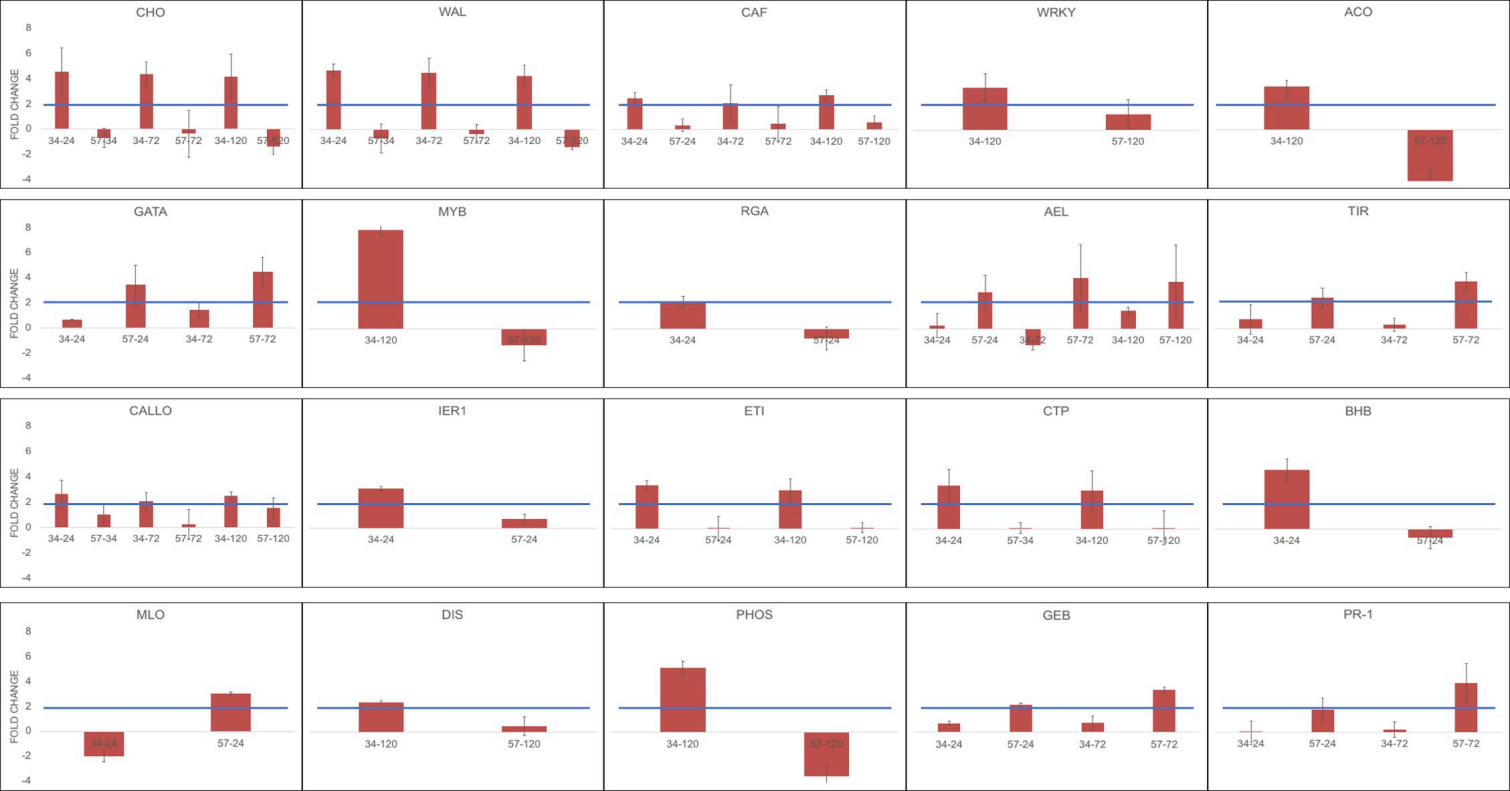
qRT-PCR validation of candidate genes. Each graph represents the fold change obtained by qRT-PCR, including the ortet and its inoculation time according to expression profile of each gene. CHO: Chorismate synthase. WAL: Wall-associated receptor kinase. CAF: caffeoylshikimate esterase. WKRY: WRKY transcription factor 40. ACO: 1-aminocicloprppane-1-1carboxylate oxidase. GATA: GATA transcription factor. MYB: MYB transcription factor. RGA: resistance gene analog. AEL: acidic endochitinase-like. TIR: transport inhibitor response 1 like. CALLO: callose synthase. IER1: immediate early response. ETI: ethylene insensitive 3. CTP: CTP synthase. BHB Transcription factor bHLB. MLO. DIS: disease resistance protein. PHOS: 1-phospatidylinositol-3-3phosphate 5 kinase. GEB: glucan endo-1,3-beta glucosidase-like. PR-1: pathogen related.

In fact, gene expression profiles and the gene networks of the resistance ortet at every point after infection suggest the presence of a salicylic acid-mediated defense response through the phenylpropanoid pathway and aromatic compounds [23, 24]. The *Chorismate synthase* (CHO) gene was overexpressed at the three studied times after infection, indicating that this resistance mechanism was present at the times the pathogen should be in the biotrophic phase, and as a result, the pathogen could not advance or cause the disease.

In the resistant ortet, a whole pathway of nitrogen compounds was upregulated, which together with the phenylpropanoid pathway and aromatic compounds suggests a defense pathway associated with cell wall reinforcement as a first defense barrier, and in the late infection processes, it could be complemented by defense pathways controlled by transcription factors such as MYB and WRKY (Fig 9).

It is important to note that the cell wall reinforcement detected in the transcriptional profile of the resistant ortet may not be associated with the degradation of the membrane components of the pathogen, as suggested by the gene expression profile of glucan endo-1,3-beta-glucosidase (GEB). This gene was found to be overexpressed (Fig 9) in the susceptible ortet, which reinforces the hypothesis that in our pathosystem, *P*. *palmivora* uses a set of highly specialized pathogenic molecules to infect oil palm tissues.

At 120 hours post infection, the resistant ortet was not only able to control the entry of the pathogen but also ensured the continuity of the redox state of the cells [12], suggesting that the stability of the redox state is a defense mechanism consistent with the distribution of genes found in the cellular component, where redox-related genes are not only expressed at the membrane level but also in the different mitochondria and plastid organelles associated with the defense response [25].

### *P*. *palmivora* molecular attack

The inoculation method used in this work did not include a physical open wound on the plant; therefore, the pathogen itself needed to find ways to colonize the tissue. This model shows that once the pathogen started its infection process, the relationship with the plant was different in the susceptible and resistant ortets. Somehow, the pathogen recognized that the ortets were different and released different mechanisms of pathogenicity.

The analyses carried out suggest that *P*. *palmivora*, in its infection process, manipulated all the transcriptional machinery of the susceptible ortet (S2 and S3 Figs) by developing fine molecular communication channels. According to reports in the literature, the successful infection process carried out by the pathogen is probably due to a greater control of the secreted effectors [26–28]; categories such as transmembrane transport activity and kinases indicate that the pathogen colonized the susceptible ortet tissue from the first hours of infection (24 hpi). The expression of these genes is consistent with the expression of genes such as MLO genes (Fig 9).

It was interesting to note that when *P*. *palmivora* attempted to colonize the resistant ortet, it continuously used genes that encode hydrolase-type proteins, and the gene distribution found in the membrane and transmembrane transport activity (S3 Fig) suggests that the pathogen recognized the membrane components of the susceptible material [29, 30].

The presence of this type of protein in the susceptible ortet suggests that it may be a response to the stimulus created by the PAMP-type molecules of the pathogen [31, 32], with which it is able to exploit the molecular mechanisms and colonize the tissue to continue its infectious process to obtain primary and secondary metabolites according to the metabolic capacity reported for oomycetes [24, 33], which is consistent with the gene expression profile of the susceptible ortet in terms of the biological process component.

The biological processes found in the gene expression of the pathogen (S3 Fig) showed how, with small differences in their transcriptional profile, the pathogen is capable of colonizing the tissue of the susceptible ortet and blocking its defense response mechanism.

The analysis of gene expression performed for the biotrophic phase of the disease shows a fine molecular communication between the palm and the pathogen, and due to a coordinated series of events, the pathogen successfully colonizes the susceptible ortet, which itself fails to develop an effective defense mechanism, while the resistant ortet blocks the spread of the pathogen.

## Supporting information

S1 Table Gene ID, primers, annealing temperature and amplicon size of genes used for qRT-PCR validation. (DOCX)

S1 Fig qRT-PCR Validation of candidate genes for *P*. *palmivora*. (TIFF)

S2 Fig GO distribution for molecular function of DEG in *P*. *palmivora*. Top panel Ortet 34 (Resistant), Bottom panel Ortet 57 susceptible.
(TIFF)

S3 Fig GO distribution for biological process of DEG in *P*. *palmivora*. Top panel Ortet 34 (Resistant), Bottom panel Ortet 57 susceptible.
(TIFF)
