# Supplementary material for: Simultaneous transcriptome analysis of oil palm clones and Phytophthora palmivora reveals oil palm defense strategies
Source: PLoS One. 2019 Sep 25;14(9):e0222774. doi: 10.1371/journal.pone.0222774 (PMC6760804; doi:10.1371/journal.pone.0222774)
Supplement: S1 Table — (DOCX) [file pone.0222774.s001.docx]

| GenID | Primer Name | Forward (3` - 5`) | Reverse (3` - 5`) | Annealing | Amplicon |
| --- | --- | --- | --- | --- | --- |
| Eg14_g010350 | CHO | TTGAGTTCGGCAGTGGATTT | CATTGGTTATCCCTCCCTGTATG | 59 | 135 |
| Eg11_g000710 | WAL | GCTTGGCCCTTCTACTCTTAC | CTCCATGTCGCCGAAAGTAT | 59 | 99 |
| Eg12_g002670 | CAF | CAATGGTCCTTCTCCTCCATAG | CAATGGGTGAGGCTTCATTTC | 60 | 104 |
| Eg04_g024000 | WRKY | GTCAGAAGGTGACCAGAGATAAC | GCACTTCTTTGCACCTTCTTC | 60 | 97 |
| Eg08_g012810 | ACO | TGGGAATAGGATGTCGATTGC | CTTCCTCTCTGCTTCTTTCTCC | 57 | 97 |
| Eg11_g007120 | MYB | CGCTCTGCTCACCAACTATATTC | CATCTCAGTCTGCAGCTCTTTC | 57 | 105 |
| Eg04_g002030 | GATA | TCCACTTCTCCCATCCTTCT | CTCGGTGAAGCTTAGGATGTT | 57 | 96 |
| EgUn-random_g095760 | AEL | AAGGTCGTCGAAGAAGTCTC | GCCAACTACCTCTGGGATAA | 60 | 123 |
| Eg06_g004420 | RGA | GGGTGGCAAGAAGGGAAATA | TGCAATCTCAGTGGTGAAGAG | 60 | 90 |
| Eg04_g002650 | TIR | GCATTGCTAGCTTAGAAGAGGA | CCTATCAAGAGGCACAGCATAG | 60 | 110 |
| Eg08_g009900 | CALLO | CCTCCGGCAATTATGGAACT | TAGACGCTGATCGTACTCTAGG | 60 | 97 |
| EgUn_random_g124950 | PR-1 | GTACGGTGAGAACCTCTTCTG | GTGTTGGTGCTGTAGTCGTA | 60 | 105 |
| Eg15_g015620 | IER1 | GCAGCTCATAGGGCTTATCTATG | GACTCAGCCTGAAACCAACT | 60 | 100 |
| Eg08_g003440 | CTP | CCATTAGACTCCGCCTTCTTT | CACCCTCTCTGTCTCTCTCTAA | 60 | 95 |
| Eg04_g024650 | ETI3 | GATCTGGGTATTCCTGCTGATG | TCCCTGGTTCTTGTTAGGATTG | 59 | 90 |
| Eg09_g008080 | PHOS | GGTTGCTTGCTTCCGTTATG | CTCATTCGCCTCCTTCTGTATC | 60 | 112 |
| Eg02_g011620 | BHB | GATGAAGCTCCTCCAAGAACTG | GAGGGACTGTACATGGTTGATG | 60 | 94 |
| Eg08_g013050 | DIS | CTGGCTTAGGAGTGAGAGATTG | ATTTGGCCGATCAGGTAGTG | 60 | 104 |
| Eg13_g003470 | MLO | CCTCATCCACTTTGTCCTCTTC | CTGGTGGAAGCATGAAGGAA | 60 | 94 |
| EgUn_random_g049390 | GEB | TGTTGTAACAGACGGGCAATA | CATTAGACCCTCCAACCTTCTC | 60 | 95 |
| PpalZC1_00000025-RA | RIB | CTCAAGCCGCACAAGTT | CGCTTCTCATAGTTGGTGTAG | 60 | 89 |
| PpalZC1_00032933-RA | ELI | GACTCGGGTTACTCTCCGTCTA | TGTAGTCACTGGCGTGATGAG | 60 | 174 |
| PpalZC1_00000691-RA | PRO | TGGTTGGGGTCTTGTGACAG | TTCGAAGCTGTGCAGTCGAT | 60 | 165 |
| PpalZC1_00005681-RA | SPO | ATCAGAATCACGGCAAGTCC | CGCCACTGGTCTTCCTTTTA | 60 | 168 |
| PpalZC1_00006753-RA | THIO | GACGACATGATCGAGGAGCA | ACTGTCCACGAAGACGAGCA | 60 | 192 |
