# Supplementary figures and images for: Simultaneous transcriptome analysis of oil palm clones and Phytophthora palmivora reveals oil palm defense strategies
Source: PLoS One. 2019 Sep 25;14(9):e0222774. doi: 10.1371/journal.pone.0222774 (PMC6760804; doi:10.1371/journal.pone.0222774)

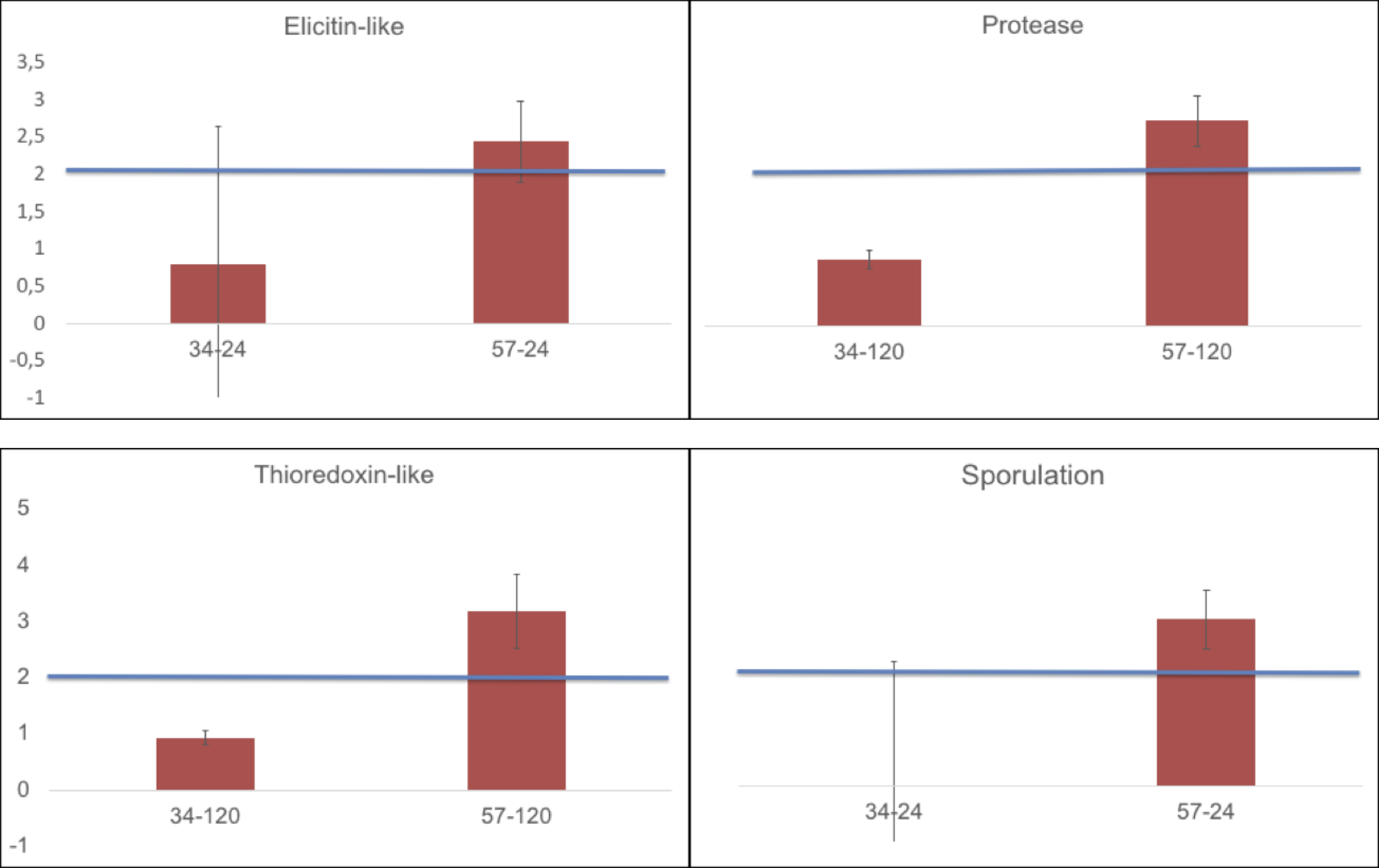

Supplement: S1 Fig — (TIFF) [file pone.0222774.s002.tiff]
